# High expression of *SOMATIC EMBRYOGENESIS RECEPTOR-LIKE KINASE* coincides with initiation of various developmental pathways in in vitro culture of *Trifolium nigrescens*

**DOI:** 10.1007/s00709-015-0814-5

**Published:** 2015-04-16

**Authors:** Maria Pilarska, Przemysław Malec, Jan Salaj, Filip Bartnicki, Robert Konieczny

**Affiliations:** The Franciszek Górski Institute of Plant Physiology, Polish Academy of Sciences, Niezapominajek 21, 30-239 Kraków, Poland; Department of Plant Physiology and Biochemistry, Faculty of Biochemistry, Biophysics and Biotechnology, Jagiellonian University, Gronostajowa 7, 30-387 Kraków, Poland; Institute of Plant Genetics and Biotechnology, Slovak Academy of Sciences, Akademicka 2, 950-07 Nitra, Slovak Republic; Department of Plant Cytology and Embryology, Institute of Botany, Jagiellonian University, Gronostajowa 9, 30-387 Kraków, Poland

**Keywords:** Gene expression, Differentiation, Pluripotency, Somatic embryogenesis, Totipotency, Transdifferentiation

## Abstract

The aim of this study was to identify and examine the expression pattern of the ortholog of *SOMATIC EMBRYOGENESIS RECEPTOR-LIKE KINASE* gene from *Trifolium nigrescens* (*TnSERK*) in embryogenic and non-regenerative cultures of immature cotyledonary-stage zygotic embryos (CsZEs). In the presence of 1-naphthaleneacetic acid and N^6^-[2-isopentenyl]-adenine, the CsZE regenerated embryoids directly and in a lengthy culture produced callus which was embryogenic or remained non-regenerative. As revealed by semi-quantitative reverse transcription polymerase chain reaction (RT-PCR), the *TnSERK* was expressed in both embryogenic and non-regenerative cultures, but the expression level was significantly higher in embryogenic ones. An in situ RNA hybridization assay revealed that the expression of *TnSERK* preceded the induction of cell division in explants, and then, it was maintained exclusively in actively dividing cells from which embryoids, embryo-like structures (ELSs), callus or tracheary elements were produced. However, the cells involved in different morphogenic events differed in intensity of hybridization signal which was the highest in embryogenic cells. The *TnSERK* was up-regulated during the development of embryoids, but in cotyledonary embryos, it was preferentially expressed in the regions of the apical meristems. The occurrence of morphological and anatomical abnormalities in embryoid development was preceded by a decline in *TnSERK* expression, and this coincided with the parenchymatization of the ground tissue in developing ELSs. *TnSERK* was also down-regulated during the maturation of parenchyma and xylem elements in CsZE and callus. Altogether, these data suggest the involvement of *TnSERK* in the induction of various developmental programs related to differentiation/transdifferentiation and totipotent state of cell(s).

**Electronic supplementary material:**

The online version of this article (doi:10.1007/s00709-015-0814-5) contains supplementary material, which is available to authorized users.

## Introduction

Somatic embryogenesis (SE) is a process whereby a single plant cell or group of cells from somatic tissue forms an embryo. SE is commonly exploited as a model for studying the structural and molecular events which underlie plant differentiation. In recent years, extensive research has been carried out on genes with a specific role in the induction and maintenance of SE (for a review, see Ikeda et al. 2006; Elhiti et al. 2013). One of such genes is *SOMATIC EMBRYOGENESIS RECEPTOR-LIKE KINASE 1* (*SERK1*) which encodes a transmembrane protein kinase belonging to the family of leucine-rich repeat protein receptor-like kinases (Hecht et al. 2001). The *SERK* gene was initially isolated from carrot embryogenic callus where a high expression of it marked cells subsequently developing into somatic embryos (Schmidt et al. 1997). In *Arabidopsis thaliana*, five genes belonging to the *SERK* family were identified and overexpression of *SERK1* was reported to lead to a significant increase in embryogenic competence in transgenic lines (Hecht et al. 2001). So far, apart from carrot and *A. thaliana*, the *SERK1* gene has been found to be expressed during the induction of SE in monocotyledonous and dicotyledonous species, i.e. *Dactylis glomerata* (Somleva et al. 2000), *Ocotea catharinensis* (Santa-Catarina et al. 2004), *Theobroma cacao* (de Oliveira Santos et al. 2005), *Vitis vinifera* (Schellenbaum et al. 2008), *Cocos nucifera* (Pérez-Núňez et al. 2009) or *Ananas comosus* (Ma et al. 2012). Although the precise expression pattern varies among species, these results demonstrated that in different plants, up-regulation of specific *SERKs* can be attributed to the induction of totipotency. Further studies, however, showed that *SERKs* also play a part in other developmental events such as induction and maintenance of shoot and root apical meristems during organogenesis in vitro and in vivo (Nolan et al. 2003, 2009; Thomas et al. 2004; Savona et al. 2012; Du et al. 2012), male sporogenesis (Albrecht et al. 2005), apomixes (Albertini et al. 2005) as well as in defense responses (Santos et al. 2009; Huang et al. 2009). Recent studies showed that AtSERK proteins play a crucial role in brassinosteroid signaling as co-receptors of BRASSINOSTEROID-INSENSITIVE1 (BRI1) (Albrecht et al. 2008; Gou et al. 2012). This multifunctionality of *SERKs* implies a need for a detailed expressional analysis of each new identified gene to properly determine its possible function in the biology of a particular plant.

In situ hybridization (ISH) of RNA is one of the most powerful techniques developed for localizing the expression site of a gene at the cell, tissue and organ levels. ISH has been successfully employed in analyzing the spatio-temporal pattern of *SERK1* expression in sunflower (Thomas et al. 2004), *Medicago truncatula* (Nolan et al. 2009), coconut (Pérez-Núňez et al. 2009) and, more recently, *Cyclamen persicum* (Savona et al. 2012). In the genus *Trifolium*, regeneration via SE has been described in many species (see Konieczny et al. 2010). However, most of the published reports focus on the improvement of culture conditions for efficient SE induction, plant development and acclimatization, whilst the molecular basis of somatic embryo formation still remains largely unknown. So far, in this genus, the sequence and expression has not been analyzed for any of the genes from the *SERK* family.

*Trifolium nigrescens* (Viv.) is a self-incompatible diploid (2*n* = 2*x* = 16) used as a forage legume for pasture and soil improvement (Hoveland and Evers 1995). Due to its high seed production under hard grazing and its resistance to southern root knot (*Meloidogyne incognita*) and clover cyst (*Heterodera trifolii*) nematodes (Pederson and Windham 1989), *T. nigrescens* is investigated for its potential as a germplasm for the improvement of other pasture clovers, e.g. *Trifolium repens* through interspecific hybridization (Marshall et al. 2002, 2008). Earlier, we reported that somatic embryos of *T. nigrescens* can be produced from cotyledonary-stage zygotic embryos (CsZEs) directly or via callus formation (Konieczny et al. 2010). Recently, we analyzed some structural aspects of *T. nigrescens* somatic embryo formation and the involvement of cell wall components in SE (Pilarska et al. 2013). In this study, we used a *T. nigrescens* culture system to determine if an ortholog of the *SERK* gene is present in the *T. nigrescens* genome and to gain an insight into its role in the induction and during the course of SE and callus growth. We applied ISH to reveal the expression pattern of a *T. nigrescens SERK* ortholog (*TnSERK*) during direct and indirect SE and in non-regenerative callus, and these data are supplemented with reverse transcription polymerase chain reaction (RT-PCR) analyses of the *TnSERK* expression level and the histology of SE and callogenesis.

## Material and methods

### Plant material and culture conditions

Seeds of *T. nigrescens* (Viv.) ssp. *nigrescens* were obtained from the Institute of Plant Genetics and Crop Plant Research, Gatersleben, Germany. The procedure for donor plant growth, explant preparation and culture conditions for SE induction was described previously (Konieczny et al. 2010). Briefly, SE was initiated from immature zygotic embryos (cotyledonary stage, ca. 3 mm in length) on a Murashige and Skoog (1962) basal medium supplemented with 30 g l^−1^ sucrose, 8 g l^−1^ agar (Difco, USA), 0.5 mg l^−1^ 1-naphthaleneacetic acid (NAA) and 2.0 mg l^−1^ N^6^-[2-isopentenyl]-adenine (2iP), pH = 5.7. Cultures were maintained in the 16:8-h photoperiod of light and darkness.

### Histological studies

Material for histological studies was collected after 3, 5, 7, 10 and 15 days of culture. The specimens were fixed in 4 % (*v*/*v*) glutaraldehyde in a 0.1 M phosphate buffer (pH 7.2) for 2 h at room temperature, dehydrated in a graded series of ethanol and embedded in Technovit 7100 (Heraeus Kulzer, Germany) according to the manufacturer’s instructions. Sections of 5 μm in thickness were routinely stained with a 0.1 % (*w*/*v*) aquatic solution of toluidine blue O.

### DNA extraction, amplification, cloning and sequencing

Genomic DNA was extracted from the leaves of mature plants (100 mg of the fresh weight from a 2-month-old plant) using a Genomic DNA Isolation Kit (A&A Biotechnology, Poland) according to the manufacturer’s protocol. The DNA concentration and purity were controlled spectrophotometrically. Conserved regions of *SERK1* from *A. thaliana* (GenBank accession number NM_105841.4) and *M. truncatula* (GenBank accession number AY162176.1) were used to design primers for amplifying a *SERK*-specific fragment from *T. nigrescens*. The PCR amplification (reaction final volume 50 μl) was carried out with Pwo polymerase (A&A Biotechnology, Poland), 25 ng of high-purity genomic DNA as a template and a primer pair (forward: 5′ AGCCGAAGAAGATCCAGAAGTTCA 3′ and reverse: 5′ TCCATTGGCCATGTAAGGAT 3′) with a PTC-150 MiniCycler (MJ Research, USA) and the following program: 94 °C for 1 min, 30 cycles of 94 °C for 45 s, 45 °C for 1 min, 72 °C for 2 min and 72 °C for 5 min. The PCR products were separated by electrophoresis on 2 % agarose gel and identified under UV light. The resulting PCR product, ca. 330 bp in length, was excised from the gel, purified with a Gel-Out kit (A&A Biotechnology, Poland), subcloned into pJET1.2/blunt plasmid (CloneJET PCR Cloning Kit; Fermentas Thermo Scientific, USA) and subjected to sequencing. Plasmids containing a *TnSERK* fragment inserted both in sense and antisense orientation were isolated, propagated and maintained in an *Escherichia coli* DH5α strain, using standard procedures (Ausubel et al. 1995). This *TnSERK* fragment was further used to design gene-specific primers suitable for the isolation of the partial genomic sequence of *TnSERK* and RT-PCR as well as to construct a probe for in situ hybridization.

To identify the genomic sequence downstream of the initially isolated *TnSERK* fragment, the following primer pair was used: forward: 5′ATGGAGGAGACAAAGTTCTGTGC 3′ and reverse: 5′TCATCTTGGACCAGATAATTCGAC 3′. The 1250-bp product was amplified by PCR as above, cloned and analyzed by sequencing.

### Sequence analysis

The nucleotide sequences obtained were analyzed and compared with GenBank data using the nucleotide database of Basic Alignment Search Tool (BLAST) software from the National Center of Biotechnology Information (NCBI; www.ncbi.nlm.nih.gov/BLAST) (Schaffer et al. 2001). The amino acid sequences deduced were compared by protein BLASTP (Swiss Institute of Bioinformatics; http://web.expasy.org/cgi-bin/blast+/BLAST_new.pl) with Protein Knowledgebase (UniProtKB/TrEMBL). Amino acid sequence alignment was performed using ClustalW (Chenna et al. 2003). Phylogenetic analysis was carried out with Phylogeny.fr software (Dereeper et al. 2008). Conserved regions were scanned by PROSITE (de Castro et al. 2006).

### RNA extraction and cDNA synthesis

RNA was extracted from mature leaf blades, non-regenerative callus (NRC) and embryogenic callus (EC) with somatic embryos after 14 days of culture. Total RNA extraction was carried out using the Spectrum™ Plant Total RNA Kit (Sigma-Aldrich, Germany) according to the manufacturer’s instruction. To avoid DNA contamination, samples were treated with DNase I (Fermentas Thermo Scientific, USA) for 30 min at room temperature. To produce a single-stranded cDNA population, 1 μg of total RNA was reversely transcribed using a RevertAid™ First Strand cDNA Synthesis Kit (Fermentas Thermo Scientific, USA), using the random primer technique, according to the manufacturer’s protocol.

### Analysis of *TnSERK* expression

For semi-quantitative RT-PCR, the following gene-specific primers for *TnSERK* were used (forward: 5′ GCAAGTCGCAACCGATACTT and reverse: 5′ CCACGTAAACGGAGGAGATT). Primers designed to recognize a conserved domain of *ELONGATION FACTOR-1α* gene (*EF-1α*) from *T. repens* (GenBank accession number KC710340.1) were used to amplify the *EF-1α* fragment that served as internal control (forward: 5′ ACGCTCTTCTTGCTTCCACC 3′ and reverse: 5′ GTTGTCTCCCTCAAAACCGGA 3′). The PCR reaction was performed with Pwo polymerase (A&A Biotechnology, Poland) using equal volumes of cDNA obtained from 1 μg of total RNA as a template, with the following program: 95 °C for 4 min, 27 cycles (denaturation at 95 °C for 1 min, annealing at 55 °C for 45 s, elongation at 72 °C for 1 min) and termination at 72 °C for 2 min. PCR products were separated in 2 % agarose gel stained with ethidium bromide. The band intensities were measured and quantified by densitometry using the UVP BioSpectrum Imaging System with VisionWorksLS version 6.8 software (UVP, USA). The relative *EF-1α* band intensity (a single 194-bp product) was acknowledged as 100 % (Serazin-Leroy et al. 1998). The significance of results from three biological replicates was checked by one-way ANOVA and Duncan’s test (*P* ≤ 0.05). Statistica for Windows ver. 8.0 (StatSoft, Inc., Tulsa, USA) was used.

### In situ hybridization

The material for ISH was sampled after 3, 5, 7, 10 and 15 days of in vitro culture. Samples were fixed for 2 h in 4 % (*v*/*v*) paraformaldehyde + 0.25 % glutaraldehyde (*v*/*v*) in a 0.1 M sodium phosphate buffer (pH 7.2), dehydrated in a series of alcohol solutions and embedded in Paraplast (Sigma-Aldrich, Germany). Sections of 6 μm in thickness were mounted on poly-lysine-coated slides, rehydrated and incubated 30 min at 37 °C with proteinase K to remove proteins. The pJET1.2/blunt plasmid containing the 322-bp sequence fragment of *TnSERK* (partial DNA from *T. nigrescens*) was used to synthesize both sense and antisense digoxigenin-labeled RNA probes with T7 polymerase (DIG RNA Labelling Kit SP6/T7; Roche Applied Science, Germany). Hybridization was performed overnight at 54 °C with riboprobes diluted in a hybridization buffer [50 % dextran sulfate (*w*/*v*), 50 % deionized formamide (*v*/*v*), 1 % Denhardt’s solution (*v*/*v*), 5 M NaCl, 10 μg μl^−1^ tRNA, 20 μg μl^−1^ polyA]. After posthybridization washes in a saline sodium citrate (SSC) buffer (2×, 1× and 0.5× SSC) at 50 °C, the slides were treated with RNAse A (100 μg ml^−1^) for 60 min at 37 °C in order to remove all non-hybridized RNA. Then, sections were washed in an SSC buffer and incubated 30 min in a blocking solution [100 mM Tris-HCl, pH 7.5, 150 mM NaCl, 2 % bovine serum albumins (*w*/*v*), 0.3 % Triton X-100 (*v*/*v*)] and 120 min with an anti-DIG antibody coupled to alkaline phosphatase (diluted 1:200; Roche Applied Science, Germany). After washing, the sections were incubated in a solution of *p*-nitro blue tetrazolium/5-bromo-4-chloro-3-indolyl phosphate (Roche Applied Science, Germany) to develop the staining reaction. The purple precipitates were observed under an Axioplan 2 Zeiss microscope and photographed using a CCD camera (SONY DXC-S500).

## Results

### Cloning of *TnSERK* partial genomic sequence

Using high-purity genomic DNA from *T. nigrescens* as a template, a PCR with primers complementary to conserved regions in the ninth exon of the *MtSERK1* and *AtSERK1* genes enabled a successful amplification of 330-bp product. BLASTN analysis revealed the high similarity of this nucleotide sequence to corresponding regions of known plant *SERK* genes deposited in the NCBI database. In particular, the highest similarity was found to *SERK1* orthologs in Fabaceae: *Medicago sativa MsSERK1* (95 %; GenBank accession number EU421842.1), *M. truncatula MtSERK1* (93 %; GenBank accession number AY162177.1 and AY162176.1) and *Glycine max GmSERK1* (88 %; GenBank accession number NM_001251345.1). The 278-bp-long partial sequence from *T. nigrescens* was designated *TnSERK1* and deposited in the GenBank database under accession number GU139549.1.

Subsequent PCR with a new pair of primers, including a forward primer complementary to the 5′ end of a known *TnSERK* fragment and a reverse primer complementary to a conservative domain present in a known plant *SERKs* at the end of the coding region, resulted in the amplification and cloning of a 1250-bp-long genomic fragment of *TnSERK*. On a nucleotide level, this gene fragment showed the highest identity with the region of 2880–4133 bp of *MtSERK1* (91 %; see Online Resource 1) as well as with *MsSERK1* and *GmSERK1* (data not shown). An analysis performed with BLASTP vs. the UniProtKB protein database revealed the high similarity (*E* value ~0.0) of the deduced TnSERK amino acid sequence (339 residues) with C-terminal parts of over 100 known plant proteins representing SERKs. The alignment of the deduced TnSERK protein with selected sequences that produced the highest identity is presented in Online Resource 2. It shows that the primary structure of putative TnSERK corresponds closely to that found in SERK proteins from other plant species. The high level of amino acid homology observed confirms that the cloned *TnSERK* fragment covers exons 9, 10 and 11 conserved in other *SERKs*, which encode subdomains of the catalytic Ser/Thr kinase domain (Nolan et al. 2003). In fact, the PROSITE scan demonstrated the presence of both the protein kinase ATP-binding region signature between residues 25–47 and the serine/threonine protein kinase active-site signature between residues 142–154 in the predicted amino acid TnSERK sequence (not shown).

**Fig. 1:**
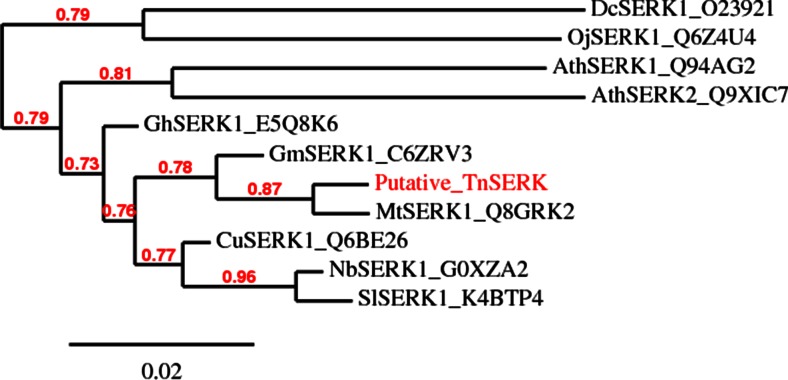
Phylogenetic relationship of TnSERK and representative plant SERKs. The phylogram was generated using the neighbour-joining method with the Phylogeny.fr program. The putative TnSERK is clustered together with MtSERK1 (*Medicago truncatula*) and GmSERK1 (*Glycine max*). Bootstrap values and a relative distance are indicated

To compare the similarity between TnSERK and SERKs from other plant species, an unrooted phylogenetic tree was constructed based on the amino acid sequence of the putative TnSERK and those of selected plants. As shown in Fig. 1, TnSERK is clustered with MtSERK1 and closely associated with GmSERK1.

### Semi-quantitative RT-PCR showed the highest expression level of *TnSERK* in embryogenic callus

**Fig. 2:**
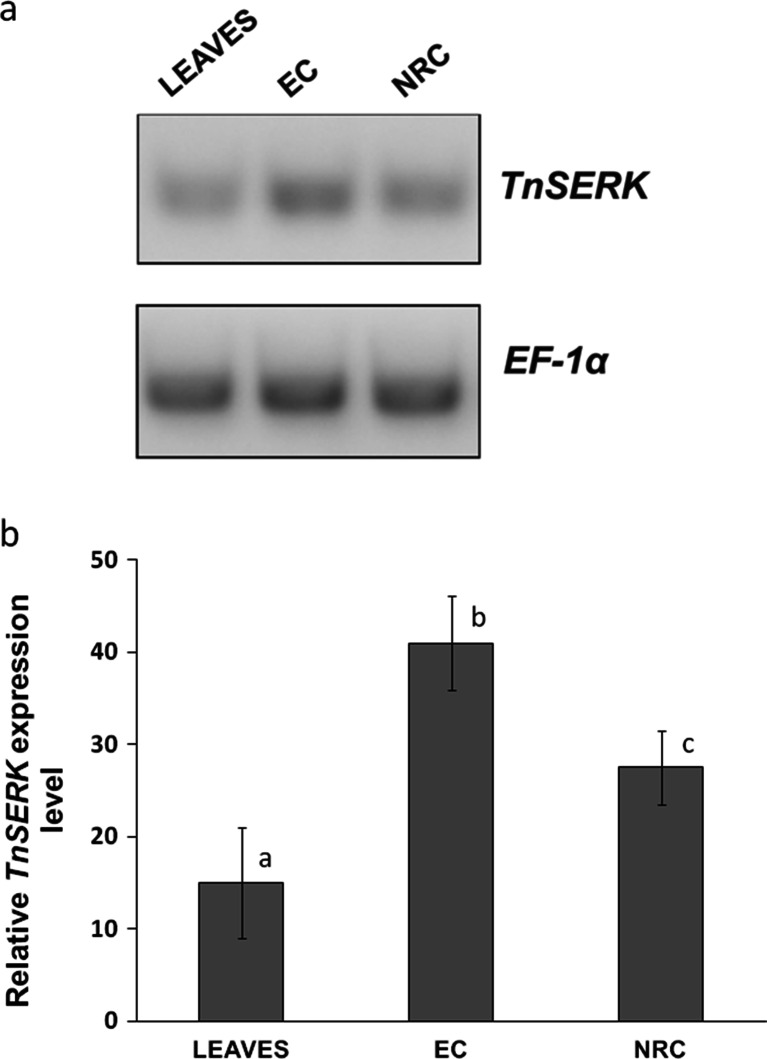
Semi-quantitative RT-PCR analysis of *TnSERK* expression in different tissues. **a** Gel image of RT-PCR products obtained with *SERK* and *EF-1α* gene-specific primers. **b** Relative expression of *TnSERK* gene after densitometric analysis. Accumulation of *EF-1α* was used as an internal control and acknowledged as 100 % in each sample type. Treatments bearing *different letters* differ significantly (*p* ≤ 0.05) by Tukey’s multiple range test. Total RNA was extracted from embryogenic callus with somatic embryos (EC), non-regenerative callus (NRC) and leaves

To examine the relative expression level of the *TnSERK* gene, total RNA was extracted from the EC, NRC and leaves of mature plants. cDNA obtained from equal amounts of total RNA was then amplified with *TnSERK*-specific primers as well as with primers specific to the housekeeping gene, *EF-1α*. The PCR conditions, including annealing temperatures and number of cycles, were optimized to avoid non-specific amplification and to determine the exponential phase of amplification (data not shown). Under our experimental conditions, the *EF-1α* transcript accumulated to a similar level in all tissue types analyzed (EC, NRC, leaves). In contrast, the relative expression of *TnSERK* showed statistically significant differences (Fig. 2a, b). The highest level of expression of *TnSERK* was observed in EC, where it was about 35 % higher than that in NRC. In mature leaves, *TnSERK* was expressed at the lowest level among material examined (ca. 60 % of the expression in EC).

### *TnSERK* transcript is detected in embryogenic and non-embryogenic cells

**Fig. 3:**
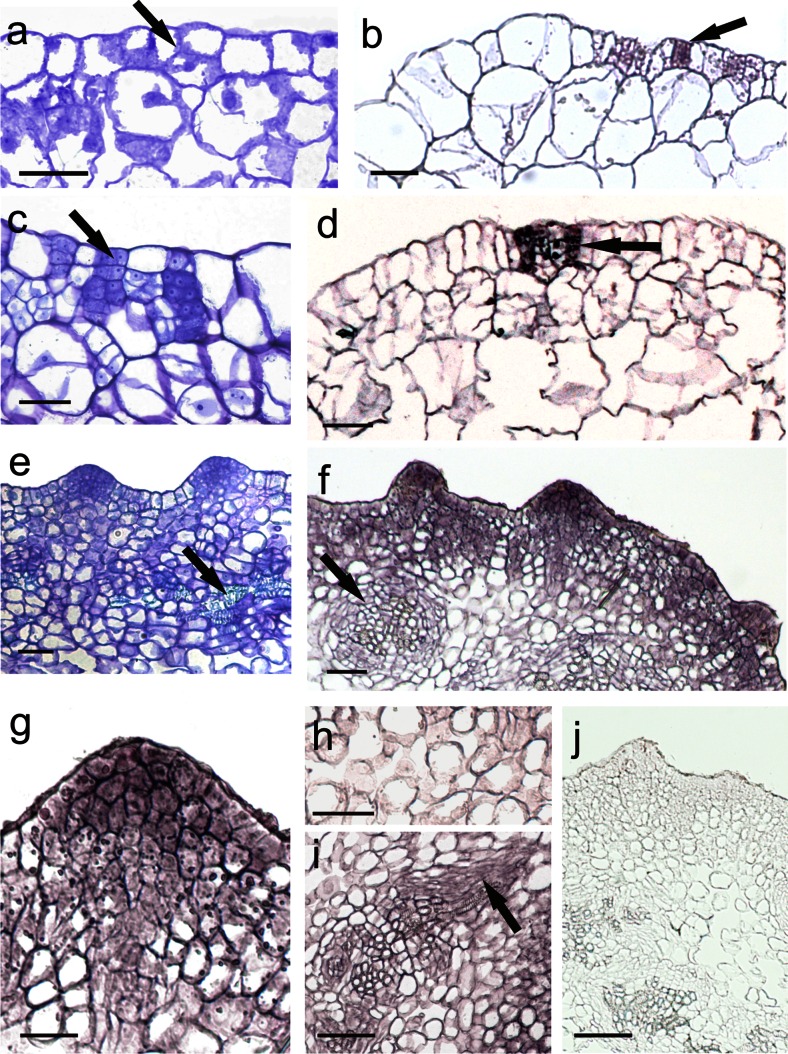
Histological analysis and the expression profiles of the *TnSERK* gene during early stages of direct SE in *T. nigrescens*. Toluidine blue-stained sections (**a**, **c**, **e**) and in situ hybridization with *TnSERK* probe (**b**, **d**, **f**–**j**). Section of hypocotyl of CsZE after 3 days (**a**, **b**), 5 days (**c**, **d**) and 7 days (**e**–**j**) of culture. **a**, **b** Small, dividing protodermal cells of explant (**a**, *arrow*) in which *TnSERK* appears (**b**, *arrow*). **c**, **d** Section of hypocotyl of CsZE showing groups of small, meristematic cells in protodermal/subprotodermal region (**c**, *arrow*) with *TnSERK* expression (**d**, arrow). **e**–**j** Explant with meristematic swellings at the peripheral part of CsZE hypocotyl; **e** xylogenic strands visible in the inner parenchyma of CsZE hypocotyl (*arrow*); **f** a strong signal of the *TnSERK* transcript localized in the cells of embryogenic swellings and cells in the subprotodermal part of explant with weaker expression visible in inner cortex and xylogenic nodules (arrow); **g** detailed view from **f** showing meristematic cells of swellings with strong hybridization signal; **h** details of cortex cells from **f** with very low expression of *TnSERK*; **i** details of xylogenic nodule showing hybridization signal in cambial-like cells adjoining tracheary elements (*arrow*); **j** section after hybridization with control *SERK* sense probe. *Bars* = 50 μm (**a**–**d**, **h**, **i**) and 100 μm (**e**, **f**, **g**, **j**)

The onset of direct SE became evident on the 3rd day in culture with the induction of periclinal division in some protodermal cells of CsZE hypocotyl (Fig. 3a). At this time, no *TnSERK* transcript in cortical cells was detected, whilst the protodermal cells were heterogenous with respect to *TnSERK* expression; along the cells lacking a *TnSERK* hybridization signal, there were single ones showing strong expression of *TnSERK* (Fig. 3b). After 5 days of culture, mitotic activity in protodermal/subprotodermal region of hypocotyl gave rise to numerous few-celled clusters consisting of small meristematic cells (Fig. 3c) with strong *TnSERK* expression (Fig. 3d). These cell clusters were maintained to day 7 followed by the formation of a few layers of meristematic tissue at the periphery of hypocotyl. Next, at the periphery of hypocotyl, numerous cone- or dome-shaped protrusions were produced (Fig. 3e). Histologically, the cells of these protrusions differed from subtending cells of a meristematic nature by having a more densely stained nucleus and a cytoplasm and a much stronger signal of *TnSERK* expression. This was apparent in the cytoplasm and nucleus and at the plasma membrane-cell wall interface (Fig. 3f, g). The hybridization signal was either very weak or not there in the non-dividing cells of the inner cortex of the CsZE (Fig. 3h). Along with formation of meristematic protrusions at the periphery of the CsZE, some xylogenic nodules randomly distributed in inner regions of the explant were also produced (Fig. 3f). The expression of *TnSERK* was not detected in the vascular core, but in the elongated cambial-like cells adjoining the tracheary elements (Fig. 3i). The use of a sense *TnSERK* riboprobe did not reveal any unspecific signal throughout the explant (Fig. 3j).

**Fig. 4:**
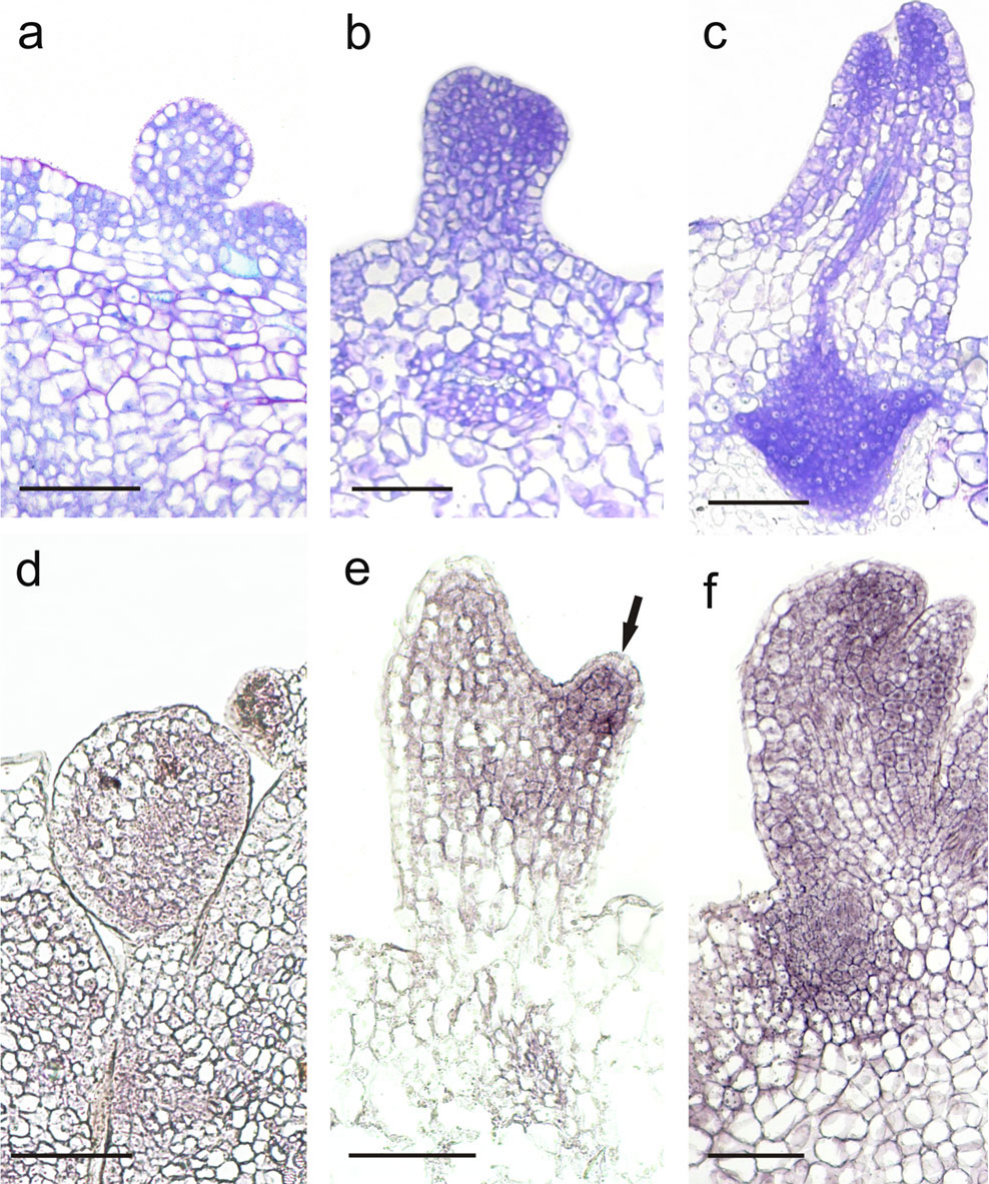
Histological analysis and the expression profiles of the *TnSERK* gene during late stages of SE in *T. nigrescens*. Toluidine blue-stained sections (**a**–**c**) and in situ hybridization with *TnSERK* probe (**d**–**f**). Somatic embryo at globular (**a**, **d**), pre-torpedo (**b**), torpedo (**e**) and cotyledonary stage (**c**, **f**). **d** Globular somatic embryo with uniform expression of *TnSERK*. **e** Intense hybridization signal visible in the cotyledon (*arrow*). **f** Mature embryo with high expression of *TnSERK* in cotyledons and root pool. *Bars* = 100 μm (**a**–**f**)

Globular embryoids differentiated directly from the protodermal/subprotodermal cells of meristematic protrusions after 10 days of culture. SE was asynchronous, and embryos at different stages of development were observed at this time of culture. The embryoids were broadly attached to the mother tissue and covered by the epidermis of the initial explant (Fig. 4a). The embryoids developed according to zygotic embryogenesis and finally formed differentiated separate cotyledonary primordia, distinct root and shoot meristems and provascular strands passing to two cotyledons (Fig. 4a–c). ISH analysis revealed that the *TnSERK* mRNA was uniformly distributed, and there were no differences in the strength of the hybridization signal between different cells of globular embryos (Fig. 4d). Further development of globular embryos was accompanied by a differential expression pattern of *TnSERK*, in which a more abundant accumulation of the *TnSERK* transcript was observed in cotyledons and at the shoot and root pole than in the ground tissues of the embryo axis (Fig. 4e, f).

### *TnSERK* is down-regulated during maturation of embryo-like structures

**Fig. 5:**
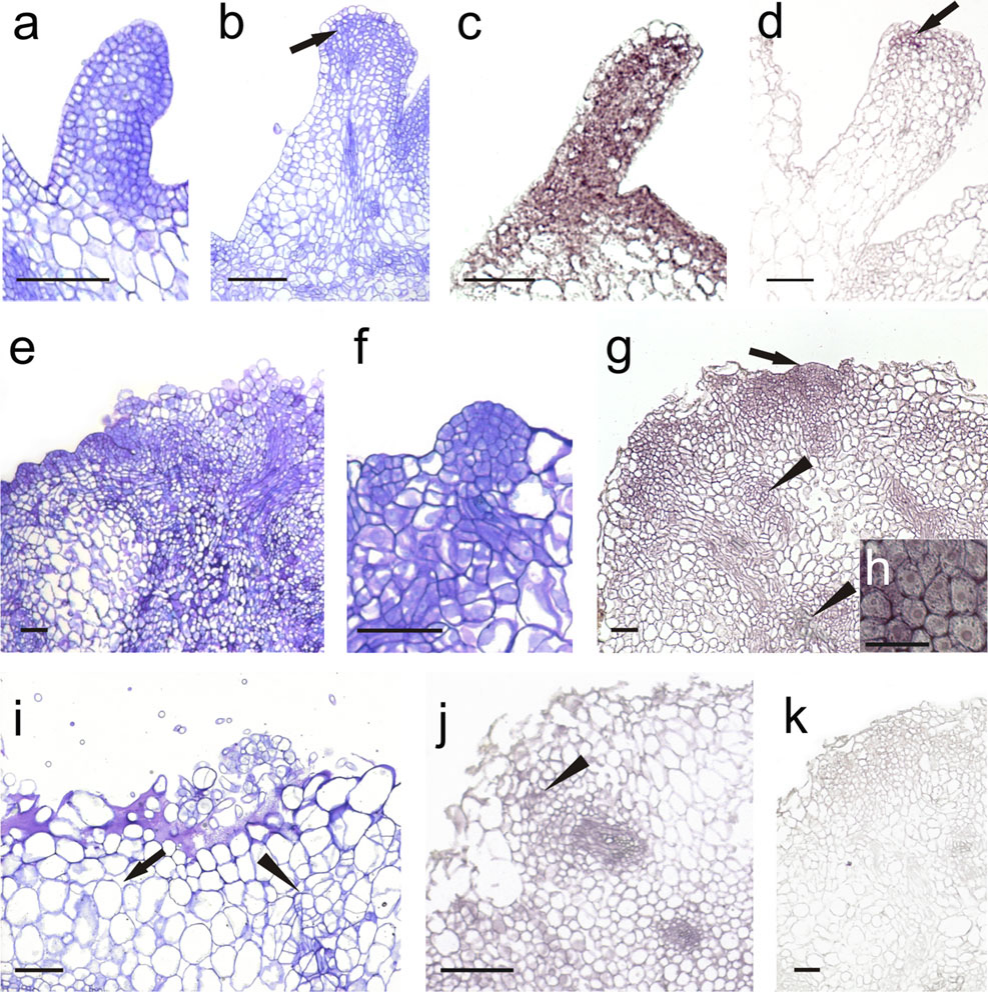
Histological analysis and *SERK1* gene expression studies in ELS and callus in *T. nigrescens*. Toluidine blue-stained sections (**a**, **b**, **e**, **f**, **i**) and ISH with *TnSERK* probe (**c**, **d**, **g**, **h**, **j**, **k**). **a**–**d** ELS formed directly on hypocotyl of CsZE; **a** emerging ELS; **b** developed ELS with meristematic cells restricted to its tip; **c** early-staged ELS with strong and uniformly distributed hybridization signal; **d** mature ELS showing hybridization signal only in the meristematic cells at its tip (*arrow*). **e** EC with meristematic multi-celled clumps localized at the periphery. **f** Early globular embryo developing indirectly. **g** EC showing *TnSERK* expression in the meristematic, embryogenic clumps (*arrow*) and xylogenic strands and nodules (*arrowhead*). **h** Detail of **g** showing intense hybridization signal in meristematic cell clumps of EC. **i** NRC composed of parenchymatic cells (*arrow*) and smaller, proliferating cells (*arrowhead*). **j** Small cells of NRC with more intense hybridization signal (*arrowhead*) and parenchymatic callus cells displaying weak hybridization signal. **k** No hybridization signal after using the sense *TnSERK* probe. *Bars* = 100 μm (**a**–**g**, **i**–**k**) and 50 μm (**h**)

Aside from somatic embryos of typical morphology, in embryogenic cultures of CsZEs, embryo-like structures (ELSs) were also produced (Online Resource 3). Embryo-like structures were initiated as elongated meristematic outgrowths, but with age, their cells enlarged and the cytoplasm and nucleus became only faintly stained or even unstained with toluidine blue (Fig. 5a, b). Cells meristematic in appearance were sometimes found at the tip of actively growing ELS, but shoot and root meristems were never formed (Fig. 5b). ISH analysis confirmed a strong signal of *TnSERK* expression, which was uniformly distributed throughout early-staged ELS, whilst in mature ones, the hybridization signal was either not detected or restricted to the group of meristematic cells at the tip of the regenerated structure (Fig. 5c, d). Mature ELSs were rod like in appearance; cotyledons were not produced. They did not develop into true organs or embryos, and with continued culture, they became necrotic and decayed.

### *TnSERK* is expressed in both embryogenic and non-regenerative callus

After 10 days of culture, direct SE was accompanied by local rupture of the CsZE epidermis and an outgrowth of callus on the surface of the explant. On 15 day, the EC was composed of compact meristematic tissue interspersed with large parenchymatous cells with the cytoplasm and nucleus undetectable by toluidine blue. Meristematic cells at the periphery of EC were mostly organized into multi-celled clumps (Fig. 5e) from which somatic embryos were induced (Fig. 5f). ISH analysis revealed a strong signal of *TnSERK* expression in all the meristematic cells of EC, but the intensity of hybridization was the strongest in peripherally located ones. The *TnSERK* transcript was confirmed either in the cytoplasm (meristematic cells of callus except of multi-celled clumps) or in the cytoplasm and nucleus and at the cell wall-plasma membrane interface (multi-celled clumps) (Fig. 5g, h). Large, parenchymatous cells at the periphery of EC and, also, these located inside the callus lacked any detectable *TnSERK* riboprobe hybridization signal (Fig. 5g). The development of callus-derived somatic embryos and ELS as well as spatio-temporal pattern of *TnSERK* expression within the embryo body during successive stages of differentiation were similar to those described above for direct SE (data not shown).

The formation of NRC was observed from CsZEs which did not display direct SE. Unlike EC, the NRC consisted mostly of large, loosely attached cells of a parenchymatous nature (Fig. 5i). The inner regions of callus contained small groups of relatively small cells with the cytoplasm somewhat more darkly stained than that of adjacent parenchyma (Fig. 5i). The hybridization signal was very weak or undetectable in the non-dividing parenchymatous cells of NRC, whilst small cells, more meristematic in appearance, showed the expression of *TnSERK* in both the cytoplasm and nucleus (Fig. 5j). Organogenesis or SE within NRC was not observed.

Within the time of culture, xylogenic nodules/strands were produced in both EC and NRC. The expression pattern of *TnSERK* in callus-derived xylogenic nodules was similar to that produced in the original explant during direct SE (Fig. 5j). When the sense *TnSERK* riboprobe was used, no signal was detected in any region of callus (Fig. 5k).

## Discussion

### *TnSERK* shows sequence similarity to other SERK proteins

In this study, for the first time, we have identified a sequence in the *T. nigrescens* genome that shows a similarity to genes encoding SERK proteins, e.g. *MtSERK1*. The members of SERK family were recently found to be both structurally related and involved in the regulation of plant responses to various biotic and abiotic stimuli (Karlova et al. 2006; Santos and Aragão 2009). Further analysis of the amino acid sequence deduced revealed a high identity of the cloned *T. nigrescens* fragment to known plant *SERKs*’ C-terminal coding region, including predominantly *MtSERK1* (Nolan et al. 2003) and *GmSERK1* (Yang et al. 2011). This result also suggests that corresponding regions of the putative *TnSERK* would play a similar role. In particular, the protein kinase active-site signature was identified in the putative TnSERK primary structure. The near 100 % identity of the corresponding region in TnSERK with the MtSERK1 indicates that this region in both proteins serves the same function. Taken together, the similarity between TnSERK and MtSERK1 in terms of amino acid structure suggests that *TnSERK* is an ortholog of the SERK1 kinases present in other plant species. Phylogenetic analysis based on predicted amino acid sequences also supports the ortholog interpretation.

### High expression of *TnSERK* accompanies induction of SE

Previously, we reported that the CsZEs of *T. nigrescens* can produce somatic embryos by culturing on media containing different combinations of auxins and cytokinins: 2,4-D and kinetin or NAA and 2iP (Konieczny et al. 2010). In this study, we used histological and ISH approaches to reveal the pattern of *TnSERK* expression in CsZEs and CsZE-derived callus maintained in the presence of NAA and 2iP. In agreement with an earlier report (Konieczny et al. 2010), the embryogenic response of CsZEs was efficient and initially confined to the hypocotyl of explants followed by the formation of hypocotyl-derived embryogenic callus. In *T. nigrescens*, the cells from which somatic embryos were differentiated displayed a number of common features, such as small size, dense cytoplasmic content and large nuclei with prominent nucleoli, and also strong expression of *TnSERK*. This was also confirmed in the SE of several dicots and monocots, e.g. *D. glomerata* (Somleva et al. 2000), *A. thaliana* (Salaj et al. 2008), *M. truncatula* (Nolan et al. 2009) or *C. persicum* (Savona et al. 2012). In some species, like carrot (Schmidt et al. 1997) or *A. thaliana* (Salaj et al. 2008), the expression of *SERK1* was found to mark the competence of single cells to follow an embryogenic pathway. The results of our study are the first to show the up-regulation of a gene from the *SERK* family in the initial cells of SE in clovers. However, the expression of *TnSERK* in culture was not only related to the production of embryoids; cells showing accumulation of the *TnSERK* transcript were also involved in xylogenesis as well as in the induction and proliferation of callus. A comparison of hybridization signal intensity in cells differing in morphogenic potential revealed, however, that cells committed to SE were more abundant in the *TnSERK* transcript than in the non-embryogenic cells of CsZE or callus. Thus, whilst the up-regulation of *TnSERK* seems to underlie different morphogenic events in *T. nigrescens*, the expression level of this gene is strongly developmentally regulated and possibly confers specific morphogenic competence of a particular cell.

### Differential pattern of *TnSERK* expression accompanies development of embryoids and embryo-like structures

Once induced, the expression of *TnSERK* continued throughout SE. As in somatic embryos of *M. truncatula* (Nolan et al. 2009), all cells of embryoids in *T. nigrescens* expressed *SERK*. However, with embryoid maturation, the pattern of gene expression changed from uniform in globular embryos to tissue-specific in cotyledonary-staged ones. In these advanced embryoids, the *TnSERK* was preferentially up-regulated in meristematic cells in shoot and root poles, which supports the notion of the involvement of *SERK* in defining and maintaining apical meristems in developing somatic embryos (Savona et al. 2012). Indeed, the ELSs which were regularly induced in the culture of *T. nigrescens* did not produce either shoot or root meristems in mature form and most of their cells lacked detectable *TnSERK* mRNA. Although a zone of meristematic cells showing a weak signal of *TnSERK* riboprobe hybridization occurred at the tip of some developing ELSs, this seems to be the result of unorganized cell proliferation rather than a step towards organized structure.

In our experimental system, both ELSs and normal zygotic-like embryoids were initiated from small, densely stained nucleus and rich in *TnSERK* transcript cells. However, unlike zygotic-like SE, abnormal development and eventual ELS formation were associated with a decline in *TnSERK* expression, and this was accompanied by the parenchymatization of ground tissue in the developing structure. In the literature, there are only sparse data on the expression of *SERK* genes in ELSs. In a culture of mangosteen, there were globular structures which did not develop into true somatic embryos (Rohani et al. 2012). These were identified as embryoids based on the presence of the *SERK1* transcript, but the expression of this gene was not monitored beyond the globular stage. In *T. nigrescens*, the occurrence of morphological and anatomical abnormalities in somatic embryos has already been reported and ascribed to a disturbance of polar auxin transport (PAT) in an early stage of development, when cotyledon primordia and embryo polarity are induced (Konieczny et al. 2012). In that study, ELSs were induced in the presence of auxinic herbicide, 2,4-D, and they shared several features, such as an elongated embryo axis, excessive parenchymatization and a lack of apical meristems, like the ELSs observed in the current experiments. The relationships between *SERKs* and PAT are only poorly understood. Du et al. (2012) revealed that a *serk1bak1bkk1* triple mutant of *A. thaliana* displayed drastically reduced expression of a number of the genes critical to PAT, cell cycle maintenance and meristem differentiation. On the other hand, different auxins were shown to up-regulate various *SERKs* (Nolan et al. 2003; Ge et al. 2010; Zhang et al. 2011). In our experimental system, the occurrence of morphological aberrations in SE preceded the decline in *SERK* expression, as the cells of early-stage ELSs displayed a very strong signal of riboprobe hybridization, typical for embryonic cells. The question as to whether the down-regulation of *TnSERK* over the further development of ELSs was related or not to the disturbance of PAT still needs to be elucidated.

### Expression of *TnSERK* meets initiation of different developmental pathways

After 10 days of culture, the CsZEs of *T. nigrescens* started to produce callus, which either regenerated somatic embryos or remained non-regenerative throughout the culture. The expression level of *TnSERK* differed according to the regenerative potential of tissue, and it was revealed by RT-PCR to be significantly higher in EC than in NRC. This is in line with studies on several species (e.g. Pérez-Núňez et al. 2009; Nolan et al. 2009; Ma et al. 2012) and adds further evidence for the involvement of *SERK* in SE in *T. nigrescens*. ISH analyses showed that aside from being expressed in embryogenic cells, *TnSERK* mRNA was also observed in populations of non-embryogenic but actively dividing cells of both EC and NRC. In contrast, the hybridization signal was either not detected or was very weak in the large and non-dividing parenchymatous cells of calluses. The dividing cells were only sparse in NRC, whilst they constituted the majority of EC which partly accounts for differences between EC and NRC in RT-PCR results. A positive correlation between the expression of *SERK* and mitotic activity was also reported in the callus of coconut (Pérez-Núňez et al. 2009) and *C. persicum* (Savona et al. 2012). Nolan et al. (2009) observed in *M. truncatula* that up-regulation of *SERK1* was not limited to dividing cells, but that it also occurred in those cells which became competent to divide. It was recently shown that callogenesis at its initial steps recapitulates the lateral root development program, and therefore, it can be interpreted as a transdifferentiation of specific somatic cell in the explant (Opatrny 2014). In a culture of *T. nigrescens*, the first cell divisions leading to morphogenesis occurred exclusively in *SERK*-expressing cells of the CsZE protoderm, and these divisions were induced de novo, as no dividing cells were found in the hypocotyl of CsZE before explantation (Konieczny et al. 2012). The expression of this gene continued in resulting mass of small actively dividing cells, but this ceased as the cell’s fate became established, e.g. in the course of the differentiation of parenchyma in developing ELSs and callus or during xylem element maturation. These observations indicate a close relationship between the level of *TnSERK* expression and the transdifferentiation/differentiation processes.

It is believed that under appropriate signal(s), plant cells can follow differentiation and transdifferentiation or acquire totipotency which manifests itself in the production of somatic embryos (Verdeil et al. 2007; Grafi et al. 2011). In *T. nigrescens*, cells expressing *SERK* were involved in different morphogenic events, but the cells of the embryogenic line showed a much stronger signal of *TnSERK* riboprobe hybridization than those cells associated with other developmental pathways, such as callus proliferation or xylogenesis. This was also observed in a culture of *C. persicum* where the initial cells for SE were said to result from pluripotentiality being maintained over time in some cells of microcallus (Savona et al. 2012). In regenerative explants of *T. nigrescens*, different morphogenic processes occurred in separate locations; SE was confined to the outermost cells of meristematic protrusions, whilst the production of meristematic but non-embryogenic cells occurred regularly in inner regions of explant/callus. This suggests a possible effect of positional information on expression level of *TnSERK* and, thereby, on cell fate. The signal(s) that would direct the peripheral and inner cells of CsZE and callus to different developmental fates remains unknown. Thorpe (1980) suggested that differences in morphogenic capacity between the inner and outer regions of the explant may reflect differences in the physiological gradient of the substances, e.g. of growth regulators from the medium into the tissues. On the other hand, it has also been pointed out that the ability of a cell to express totipotency and follow SE may be related to the removal of the embryogenesis-repressive and/or differentiation-inductive effects of neighbouring cells and tissues (Williams and Maheswaran 1986). Superficially located cells have different surroundings than those inside tissue as they are also in contact with the external environment which potentially reduces the capacity for inter-cell communication with adjacent tissues (Kurczynska et al. 2012).

In summary, the *TnSERK* gene from *T. nigrescens* was partly cloned and its expression pattern was monitored in in vitro cultured embryogenic and non-regenerative tissues. The down-regulation of *TnSERK* during cell specialization (the maturation of parenchyma and xylem elements) and its up-regulation in the actively dividing cells involved in different morphogenic processes (SE, callus production, xylogenesis, formation of apical meristems in developing embryoids) suggest broad role for it in plant development, possibly related to differentiation and transdifferentiation processes as well as induction and maintenance of totipotent state in cell(s). Unfortunately, *T. nigrescens* has not been the subject of genomic mapping or sequencing efforts so far. Consequently, the structure of *T. nigrescens* genome is unknown, which, in turn, hampers the application of molecular approaches for studying the gene function. However, an increasing interest in this species in food, agricultural and pharmaceutical industries (see: Konieczny et al. 2010, 2012; Demirkiran et al. 2013) allows for envision that the sequencing of its genome as well as the development of transformation techniques will be initiated in the nearest future.

## Electronic supplementary material

ESM 1 (PDF 182 kb)

ESM 2 (PDF 197 kb)

ESM 3 (PDF 218 kb)

## Abbreviations

2iP: N^6^-[2-isopentenyl]-adenine
CsZE: Cotyledonary-stage zygotic embryo
EC: Embryogenic callus
ELS: Embryo-like structure
ISH: In situ hybridization
NAA: 1-Naphthaleneacetic acid
NRC: Non-regenerative callus
SE: Somatic embryogenesis
*SERK*: *SOMATIC EMBRYOGENESIS RECEPTOR-LIKE KINASE*
