# Supplementary material for: High expression of SOMATIC EMBRYOGENESIS RECEPTOR-LIKE KINASE coincides with initiation of various developmental pathways in in vitro culture of Trifolium nigrescens
Source: Protoplasma. 2015 Apr 16;253:345–55. doi: 10.1007/s00709-015-0814-5 (PMC4783438; doi:10.1007/s00709-015-0814-5)
Supplement: Supplementary file 1 — (PDF 182 kb) [file 709_2015_814_MOESM1_ESM.pdf]

Electronic Supplementary Material

For manuscript entitled: “High expression of *SOMATIC EMBRYOGENESIS RECEPTOR-LIKE KINASE* coincides with initiation of various developmental pathways in *in vitro* culture of *Trifolium nigrescens*”

Submitted to *Protoplasma*

Authors: Maria Pilarska[1], Przemysław Malec[2], Jan Salaj[3], Filip Bartnicki[2], Robert Konieczny[4]*
[1]The Franciszek Górski Institute of Plant Physiology, Polish Academy of Sciences, Niezapominajek 21, 30-239 Kraków, Poland
[2]Department of Plant Physiology and Biochemistry, Faculty of Biochemistry, Biophysics and Biotechnology, Jagiellonian University, Gronostajowa 7, 30-387 Kraków, Poland
[3]Institute of Plant Genetics and Biotechnology, Slovak Academy of Sciences, Akademicka 2, 950-07 Nitra, Slovak Republic
[4]Department of Plant Cytology and Embryology, Institute of Botany, Jagiellonian University, Gronostajowa 9, 30-387 Kraków, Poland

*Author for correspondence:
e-mail: robert.konieczny@uj.edu.pl
tel/fax: +48 12 664 51 04

Online Resource 1. A nucleotide sequence alignment of 1248bp genomic fragment cloned from *Trifolium nigrescens* with *Medicago truncatula* somatic embryogenesis receptor kinase 1 (*SERK1*) gene (gb|AY162177.1) generated by BLAST

Query: Putative *TnSERK* sequence fragment (1248 bp)
Subject: *Medicago truncatula* somatic embryogenesis receptor kinase 1 (*SERK1*) gene, complete cds, Sequence ID: gb|AY162177.1|Length: 4206;

Score	Expect	Identities	Gaps	Strand
1657 bits(897)	0.0	1145/1263(91%)	24/1263(1%)	Plus/Plus

```
Query  1     GAAGAAGATCCAGAAGTTCATCTTGGGCAGCTTAAGAGATTCTCACTAAGAGAGTTGCAA
             ||||| ||||| |||||||||||||||||||||||||| ||||||||  |||||||||||
Sbjct  2880  GAAGAGGATCCTGAAGTTCATCTTGGGCAGCTTAAGAGGTTCTCACTCCGAGAGTTGCAA

Query  61    GTTGCAACAGATACTTTCAGCAATAAGAACATTCTTGGAAGAGGAGGGTTTGGTAAGGTA
             ||||||||||||||||||||||||||||||||||||||||||||||||||||| ||||| 
Sbjct  2940  GTTGCAACAGATACTTTCAGCAATAAGAACATTCTTGGAAGAGGAGGGTTTGGCAAGGTG

Query  121   TACAAAGGAAGATTGGCAGACGGTTCACTGGTTGCTGTCAAAAGGTTGAAAGAGGAGCGG
             ||||||||| | ||||| |||||||||||||||||||||||||| || ||||||||||||
Sbjct  3000  TACAAAGGACGTTTGGCTGACGGTTCACTGGTTGCTGTCAAAAGATTAAAAGAGGAGCGG

Query  181   ACACCAGGTGGGGAGCTTCAGTTTCAGACTGAAGTTGAGATGATCAGCATGGCTGTTCAT
             ||||| ||||||||||||||||||||||||||||| |||||||||||||||||||| |||
Sbjct  3060  ACACCTGGTGGGGAGCTTCAGTTTCAGACTGAAGTAGAGATGATCAGCATGGCTGTGCAT

Query  241   AGAAATCTCCTCCGTTTACGTGGGTTTTGTATGACTCCAACAGAAAGATTACTTGTTTAT
             |||||||||||||||||||| |||||||||||||| ||||| ||||| ||||| ||||||
Sbjct  3120  AGAAATCTCCTCCGTTTACGCGGGTTTTGTATGACACCAACCGAAAGGTTACTGGTTTAT

Query  301   CCATACATGGCTAATGGAAGTGTTGCCTCCTGTTTAAGAGGTATATCTCTATTTATATAT
             || |||||||||||||||||||||||||||||||||||||||| |||||||||||||| | 
Sbjct  3180  CCCTACATGGCTAATGGAAGTGTTGCCTCCTGTTTAAGAGGTAGATCTCTATTTATACA-
```

```
Query  361   ATATTGTTTGTTTATATATGTGCTCGGCATAATCTTTCATTCAATATTAATTATTATTCT
                  ||||||||||||||||||||  || ||||||||  ||||||||||||||| ||||
Sbjct  3239  -----GTTTGTTTATATATGTGCTCAACACAATCTTTCGGTCAATATTAATTATTCTTCT

Query  421   TGTGATTTTCTATTTGCATTATTCTAGAGCGCCCTCCACATCAGCAACCACTAGATTGGC
             |  |||||| | |||| ||||| |||||||| |||||||||||  |||||||||||||||
Sbjct  3294  TCCGATTTTGTGTTTGTATTATCCTAGAGCGTCCTCCACATCAAGAACCACTAGATTGGC

Query  481   CAACAAGGAAAAGAATAGCTTTGGGATCAGCAAGGGGTCTTTCATATTTGCATGATCATT
             ||||||| ||||||||||||||||||||||| ||||||||||||||||||||||||||||
Sbjct  3354  CAACAAGAAAAAGAATAGCTTTGGGATCAGCTAGGGGTCTTTCATATTTGCATGATCATT

Query  541   GTGACCCAAAGATCATTCATCGGACGTGAAAGCTGCTAACATATTGTTGGATGAAGAGT
             |||||||||| ||||||||||| |||||||||||||||||||||||||||||||||||||
Sbjct  3414  GTGACCCAAAAATCATTCATCGTGACGTGAAAGCTGCTAACATATTGTTGGATGAAGAGT

Query  601   TTGAGGCTGTTGTTGGGGATTTTGGATTGGCAAAACTTATGGATTACAAGGACACTCATG
             ||||||||||||| ||||||||||||||||||||||||||||||||||||||||| ||||
Sbjct  3474  TTGAGGCTGTTGTGGGGGATTTTGGATTGGCAAAACTTATGGATTACAAGGACACCCATG

Query  661   TGACAACTGCTGTTCGGGGTACAATAGGGCATATAGCTCCCGAGTACCTATCTACCGGAA
             | ||||||||||||||||||||||| ||||||||||||||||||||||||||||| || |
Sbjct  3534  TTACAACTGCTGTTCGGGGTACAATTGGGCATATAGCTCCCGAGTACCTATCTACTGGCA

Query  721   AATCTTCAGAGAAAACTGATGTTTTTGGTTATGGTATCATGCTTCTTGAGCTTATCACTG
             | ||||||||||||||||||||||||||||||||||||||||||||||||||||| ||||
Sbjct  3594  AGTCTTCAGAGAAAACTGATGTTTTTGGTTATGGTATCATGCTTCTTGAGCTTATAACTG

Query  781   GACAAAGAGCTTTTGACCTTGCTCGGCTTGCCAATGATGATGATGTTATGCTGCTTGATT
             ||||||||||||||||||||||||| ||||| ||||||||||||||||||||||||||||
Sbjct  3654  GACAAAGAGCTTTTGACCTTGCTCGACTTGCGAATGATGATGATGTTATGCTGCTTGATT

Query  841   GGGTATGTTCGGTGCAAATGCATATCCTTCTGATTTTCGTTTATTATATAGTTTACCTTT
             |||||||||| ||||||| ||| ||||||||| | || ||||||| | ||||||||  ||
Sbjct  3714  GGGTATGTTCAGTGCAAACGCACATCCTTCTGTTCTTTGTTTATT-T-TAGTTTACGCTT

Query  901   TCCGTTGAATTTCATT----TCATGCAACCCTTG---A---AA-T-A---TGTTTATAGG
             || ||||||||||||     || || |||  |||   |   || | |   |||| |||||
Sbjct  3772  TCAGTTGAATTTCATGCAACTCTTG-AACAATTGTGCACTCAACTGAACTTGTTCATAGG

Query  946   TAAAAGGACTTCTGAAAGAGAAAAAACTTGAAATGTTGGTTGATCCTGATCTTCAAACCA
             ||||||||||||||||||||||||| |||||||||||||||||||| |||||| ||||||
Sbjct  3831  TAAAAGGACTTCTGAAAGAGAAAAAGCTTGAAATGTTGGTTGATCCCGATCTTAAAACCA

Query  1006  ACTACATAGAAGCCGAGGTAGAACAGTTAATCCAGGTTGCACTACTCTGCACACAAGGTT
             ||||||||||||| |||||||||||||||||||||||||| || || ||||| |||||||
Sbjct  3891  ACTACATAGAAGCTGAGGTAGAACAGTTAATCCAGGTTGCGCTGCTATGCACGCAAGGTT

Query  1066  CACCTATGGACCGGCCGAAGAGGTCGGAAGTGGTGAGAATGCTTGAAGGCGACGGTTTGG
             | |||||||||||||| |||| ||| || || |||||||||||||||||||| || ||||
Sbjct  3951  CGCCTATGGACCGGCCAAAGATGTCAGATGTAGTGAGAATGCTTGAAGGCGATGGCTTGG

Query  1126  CAGAAAGATGGGACGAGTGGCAAAAGGGAGAAGTACTTCGCCAGGAAGTGGAACTCGCCC
             ||||||||||||| ||||||||||| || ||||| || ||||||||||||||| | || |
Sbjct  4011  CAGAAAGATGGGATGAGTGGCAAAAAGGGGAAGTTCTACGCCAGGAAGTGGAATTGGCAC

Query  1186  CTCATCCTAATTCGGATTGGATTGTCGACTCTACCGAAAATCTACATGCAGTCGAATTAT
             ||||||| ||||| ||||||||||| |||||||| |||||||||||||||||||||||||
Sbjct  4071  CTCATCCCAATTCTGATTGGATTGTTGACTCTACTGAAAATCTACATGCAGTCGAATTAT
```

```
Query  1246  CTG  1248
             |||
Sbjct  4131  CTG  4133
```
