# Supplementary material for: High expression of SOMATIC EMBRYOGENESIS RECEPTOR-LIKE KINASE coincides with initiation of various developmental pathways in in vitro culture of Trifolium nigrescens
Source: Protoplasma. 2015 Apr 16;253:345–55. doi: 10.1007/s00709-015-0814-5 (PMC4783438; doi:10.1007/s00709-015-0814-5)
Supplement: Supplementary file 2 — (PDF 197 kb) [file 709_2015_814_MOESM2_ESM.pdf]

Electronic Supplementary Material

For manuscript entitled: "High expression of *SOMATIC EMBRYOGENESIS RECEPTOR-LIKE KINASE* coincides with initiation of various developmental pathways in *in vitro* culture of *Trifolium nigrescens*"

Submitted to *Protoplasma*

Authors: Maria Pilarska[1], Przemysław Malec[2], Jan Salaj[3], Filip Bartnicki[2], Robert Konieczny[4]*
[1]The Franciszek Górski Institute of Plant Physiology, Polish Academy of Sciences, Niezapominajek 21, 30-239 Kraków, Poland
[2]Department of Plant Physiology and Biochemistry, Faculty of Biochemistry, Biophysics and Biotechnology, Jagiellonian University, Gronostajowa 7, 30-387 Kraków, Poland
[3]Institute of Plant Genetics and Biotechnology, Slovak Academy of Sciences, Akademicka 2, 950-07 Nitra, Slovak Republic
[4]Department of Plant Cytology and Embryology, Institute of Botany, Jagiellonian University, Gronostajowa 9, 30-387 Kraków, Poland

*Author for correspondence:
e-mail: robert.konieczny@uj.edu.pl
tel/fax: +48 12 66 51 04

Online Resource 2. Alignment of the deduced partial amino acid sequence of TnSERK with other plant SERKs: *Medicago truncatula* (Q8GRK2) *Glycine max* (C6ZRV3), *Citrus unshiu* (Q6BE26), *Gossypium hirsutum* (E5Q8K6), *Nicotiana benthamiana* (G0XZA2), *Solanum lycopersicum* (K4BTP4), *Arabidopsis thaliana* SERK1 (Q94AG2), *Arabidopsis thaliana* SERK2 (Q9XIC7), *Oryza japonica* (Q6Z4U4) and *Daucus carrota* (O23921)

```
Putative TnSERK        1    LGQLKRFSLRELQVATDTFSNKNILGRGGFGKVYKGRLADGSLVAVKRLKEERTPGGELQFQTEVEMISMAVHRNLLRLR
MtSERK1 [Q8GRK2]       1    LGQLKRFSLRELQVATDTFSNKNILGRGGFGKVYKGRLADGSLVAVKRLKEERTPGGELQFQTEVEMISMAVHRNLLRLR
GmSERK1 [C6ZRV3]       1    LGQLKRFSLRELQVATDSFSNKNILGRGGFGKVYKGRLADGSLVAVKRLKEERTPGGELQFQTEVEMISMAVHRNLLRLR
CuSERK1 [Q6BE26]       1    LGQLKRFSLRELQVATDSFSNKNILGRGGFGKVYKGRLADGSLVAVKRLKEERTPGGELQFQTEVEMISMAVHRNLLRLR
GhSERK1 [E5Q8K6]       1    LGQLKRFSLRELQVATDSFSHKNILGRGGFGKVYKGRLADGSLVAVKRLKEERTPGGELQFQTEVEMISMAVHRNLLRLR
NbSERK1 [G0XZA2]       1    LGQLKRFSLRELQVATDSFSNKNILGRGGFGKVYKGRLADGTLVAVKRLKEERTPGGELQFQTEVEMISMAVHRNLLRLR
SlSERK1 [K4BTP4]       1    LGQLKRFSLRELQVATDSFSNKNILGRGGFGKVYKGRLADGSLVAVKRLKEERTPGGELQFQTEVEMISMAVHRNLLRLR
AthSERK1 [Q94AG2]      1    LGQLKRFSLRELQVASDGFSNKNILGRGGFGKVYKGRLADGTLVAVKRLKEERTPGGELQFQTEVEMISMAVHRNLLRLR
AthSERK2 [Q9XIC7]      1    LGQLKRFSLRELQVATDSFSNKNILGRGGFGKVYKGRLADGTLVAVKRLKEERTPGGELQFQTEVEMISMAVHRNLLRLR
OjSERK1 [Q6Z4U4]       1    LGQLKRFSLRELQVATDTFSNKNILGRGGFGKVYKGRLADGSLVAVKRLKEERTPGGELQFQTEVEMISMAVHRNLLRLR
DcSERK1 [O23921]       1    LGQLKRFSLRELQVATDTFS--TILGRGGFGKVYKGRLADGSLVAVKRLKEERTPGGELQFQTEVEMISMAVHRNLLRLR
Clustal Consensus      1    ***************:* **  .******************:**************************************

Putative TnSERK        81   GFCMTPTERLLVYPYMANGSVASCLRERPPHQQPLDWPTRKRIALGSARGLSYLHDHCDPKIIHRDVKAANILLDEEFEA
MtSERK1 [Q8GRK2]       81   GFCMTPTERLLVYPYMANGSVASCLRERPPHQEPLDWPTRKRIALGSARGLSYLHDHCDPKIIHRDVKAANILLDEEFEA
GmSERK1 [C6ZRV3]       81   GFCMTPTERLLVYPYMANGSVASCLRERPPYQEPLDWPTRKRVALGSARGLSYLHDHCDPKIIHRDVKAANILLDEEFEA
CuSERK1 [Q6BE26]       81   GFCMTPTERLLVYPYMANGSVASCLRERPPSQLPLDWPTRKRIALGSARGLSYLHDHCDPKIIHRDVKAANILLDEEFEA
GhSERK1 [E5Q8K6]       81   GFCMTPTERLLVYPYMANGSVASCLRERPPSQPPLDWPTRKRIALGSARGLSYLHDHCDPKIIHRDVKAANILLDEEFEA
NbSERK1 [G0XZA2]       81   GFCMTPTERLLVYPYMANGSVASCLRERPPSEPPLDWPTRKRIALGSARGLSYLHDHCDPKIIHRDVKAANILLDEEFEA
SlSERK1 [K4BTP4]       81   GFCMTPTERLLVYPYMANGSVASCLRERPPSEPPLDWPTRKRIALGSARGLSYLHDHCDPKIIHRDVKAANILLDEEFEA
AthSERK1 [Q94AG2]      81   GFCMTPTERLLVYPYMANGSVASCLRERPPSQPPLDWPTRKRIALGSARGLSYLHDHCDPKIIHRDVKAANILLDEEFEA
AthSERK2 [Q9XIC7]      81   GFCMTPTERLLVYPYMANGSVASCLRERPPSQLPLAWSIRQQIALGSARGLSYLHDHCDPKIIHRDVKAANILLDEEFEA
OjSERK1 [Q6Z4U4]       81   GFCMTPTERLLVYPYMANGSVASRLRERPPSEPPLDWRTRRRIALGSARGLSYLHDHCDPKIIHRDVKAANILLDEDFEA
DcSERK1 [O23921]       79   GFCMTPTERLLVYPYMANGSVASCLRERQPSEPPLDWPTRKRIALGSARGLSYLHDHCDPKIIHRDVKAANILLDEEFEA
Clustal Consensus      77   *********************** **** * : ** *  *:::*********************************:***

Putative TnSERK        161  VVGDFGLAKLMDYKDTHVTTAVRGTIGHIAPEYLSTGKSSEKTDVFGYGIMLLELITGQRAFDLARLANDDDVMLLDWVK
MtSERK1 [Q8GRK2]       161  VVGDFGLAKLMDYKDTHVTTAVRGTIGHIAPEYLSTGKSSEKTDVFGYGIMLLELITGQRAFDLARLANDDDVMLLDWVK
GmSERK1 [C6ZRV3]       161  VVGDFGLAKLMDYKDTHVTTAVRGTIGHIAPEYLSTGKSSEKTDVFGYGIMLLELITGQRAFDLARLANDDDVMLLDWVK
CuSERK1 [Q6BE26]       161  VVGDFGLAKLMDYKDTHVTTAVRGTIGHIAPEYLSTGKSSEKTDVFGYGIMLLELITGQRAFDLARLANDDDVMLLDWVK
GhSERK1 [E5Q8K6]       161  VVGDFGLAKLMDYKDTHVTTAVRGTIGHIAPEYLSTGKSSEKTDVFGYGIMLLELITGQRAFDLARLANDDDVMLLDWVK
NbSERK1 [G0XZA2]       161  VVGDFGLAKLMDYKDTHVTTAVRGTIGHIAPEYLSTGKSSEKTDVFGYGIMLLELITGQRAFDLARLANDDDVMLLDWVK
SlSERK1 [K4BTP4]       161  VVGDFGLAKLMDYKDTHVTTAVRGTIGHIAPEYLSTGKSSEKTDVFGYGIMLLELITGQRAFDLARLANDDDVMLLDWVK
AthSERK1 [Q94AG2]      161  VVGDFGLAKLMDYKDTHVTTAVRGTIGHIAPEYLSTGKSSEKTDVFGYGIMLLELITGQRAFDLARLANDDDVMLLDWVK
AthSERK2 [Q9XIC7]      161  VVGDFGLARLMDYKDTHVTTAVRGTIGHIAPEYLSTGKSSEKTDVFGYGIMLLELITGQRAFDLARLANDDDVMLLDWVK
OjSERK1 [Q6Z4U4]       161  VVGDFGLAKLMDYKDTHVTTAVRGTIGHIAPEYLSTGKSSEKTDVFGYGIMLLELITGQRAFDLARLANDDDVMLLDWVK
DcSERK1 [O23921]       159  VVGDFGLARLMDYKDTHVTTAVRGTLGYIAPEYLSTGKSSEKTDVFGYGIMLLELITGQRAFDLARLANDDDVMLLDWVK
Clustal Consensus      150  ********:****************:*:****************************************************

Putative TnSERK        241  GLLKEKKLEMLVDPDLQTNYIEAEVEQLIQVALLCTQGSPMDRPKRSEVVRMLEGDGLAERWDEWQKGEVLRQEVELAPH
MtSERK1 [Q8GRK2]       241  GLLKEKKLEMLVDPDLKTNYIEAEVEQLIQVALLCTQGSPMDRPKMSDVVRMLEGDGLAERWDEWQKGEVLRQEVELAPH
GmSERK1 [C6ZRV3]       241  GLLKEKKLEMLVDPDLQTNYIETEVEQLIQVALLCTQGSPMDRPKMSEVVRMLEGDGLAERWDEWQKVEVLRQEVELAPH
CuSERK1 [Q6BE26]       241  GLLKEKKLEMLVDPDLQNNYVEAEVEQLIQVALLCTQGSPMDRPKMSEVVRMLEGDGLAERWDEWQKVEVLRQEVELAPH
GhSERK1 [E5Q8K6]       241  GLLKEKKLELLVDPDLQTNYVETEVEQLIQVALLCTQGSPMDRPKMSEVVRMLEGDGLAERWDEWQKVEVLRQEVELAPH
NbSERK1 [G0XZA2]       241  GLLKEKKLEMLVDPDLQNKYVEAEVEQLIQVALLCTQSSPMDRPKMSEVVRMLEGDGLAERWDEWQKVEVLRQEVELAPH
SlSERK1 [K4BTP4]       241  GLLKEKKLEMLVDPDLQNKYVEAEVEQLIQVALLCTQSNPMDRPKMSEVVRMLEGDGLAERWDEWQKVEVLRQEVELAPH
AthSERK1 [Q94AG2]      241  GLLKEKKLEMLVDPDLQTNYEERELEQVIQVALLCTQGSPMERPKMSEVVRMLEGDGLAEKWDEWQKVEILREEIDLSPN
AthSERK2 [Q9XIC7]      241  GLLKEKKLEMLVDPDLQSNYTEAEVEQLIQVALLCTQSSPMERPKMSEVVRMLEGDGLAEKWDEWQKVEVLRQEVELSSH
OjSERK1 [Q6Z4U4]       241  GLLKEKRLEMLVDPDLQSNYIDVEVESLIQVALLCTQGSPTERPKMAEVVRMLEGDGLAERWEEWQKIEVVRQEVELCPH
DcSERK1 [O23921]       239  SLLKEKKLEMLVDPDLENNYIDTEVEQLIQVALLCTQGSPMERPKMSEVVRMLEGDGLAEKWDEWQKVEVIHQDVELAPH
Clustal Consensus      229  .*****:**:******:.:* : *:*.:*********..* :*** ::************:*:**** *:::::::*..:

Putative TnSERK        321  PNSDWIVDSTENLHAVELS
MtSERK1 [Q8GRK2]       321  PNSDWIVDSTENLHAVELS
GmSERK1 [C6ZRV3]       321  PNSDWIVDSTENLHAVELS
CuSERK1 [Q6BE26]       321  PNSDWIVDSTENLHAVELS
GhSERK1 [E5Q8K6]       321  PNSDWIVDSTDNLHAVELS
NbSERK1 [G0XZA2]       321  PGSDWIVDSTENLHAVELS
SlSERK1 [K4BTP4]       321  PGSDWLVDSTENLHAVELS
AthSERK1 [Q94AG2]      321  PNSDWILDSTYNLHAVELS
AthSERK2 [Q9XIC7]      321  PTSDWILDSTDNLHAMELS
OjSERK1 [Q6Z4U4]       321  RNSEWIVDSTDNLHAVELS
DcSERK1 [O23921]       319  RTSEWILDSTDNLHAFELS
Clustal Consensus             *:*::*** ****.***
```
