# Supplementary material for: High expression of SOMATIC EMBRYOGENESIS RECEPTOR-LIKE KINASE coincides with initiation of various developmental pathways in in vitro culture of Trifolium nigrescens
Source: Protoplasma. 2015 Apr 16;253:345–55. doi: 10.1007/s00709-015-0814-5 (PMC4783438; doi:10.1007/s00709-015-0814-5)

Electronic Supplementary Material

For manuscript entitled: "High expression of *SOMATIC EMBRYOGENESIS RECEPTOR-LIKE KINASE* coincides with initiation of various developmental pathways in *in vitro* culture of *Trifolium nigrescens*"

Submitted to *Protoplasma*

Authors: Maria Pilarska[1], Przemysław Malec[2], Jan Salaj[3], Filip Bartnicki[2], Robert Konieczny[4]*
[1]The Franciszek Górski Institute of Plant Physiology, Polish Academy of Sciences, Niezapominajek 21, 30-239 Kraków, Poland
[2]Department of Plant Physiology and Biochemistry, Faculty of Biochemistry, Biophysics and Biotechnology, Jagiellonian University, Gronostajowa 7, 30-387 Kraków, Poland
[3]Institute of Plant Genetics and Biotechnology, Slovak Academy of Sciences, Akademicka 2, 950-07 Nitra, Slovak Republic
[4]Department of Plant Cytology and Embryology, Institute of Botany, Jagiellonian University, Gronostajowa 9, 30-387 Kraków, Poland

*Author for correspondence:
e-mail: robert.konieczny@uj.edu.pl
tel/fax: +48 12 664 51 04

Online Resource 3. Somatic embryos of zygotic-like morphology (a) and embryo-like structures induced from zygotic embryos of *T. nigrescens* (b). Bar represents 500µm.

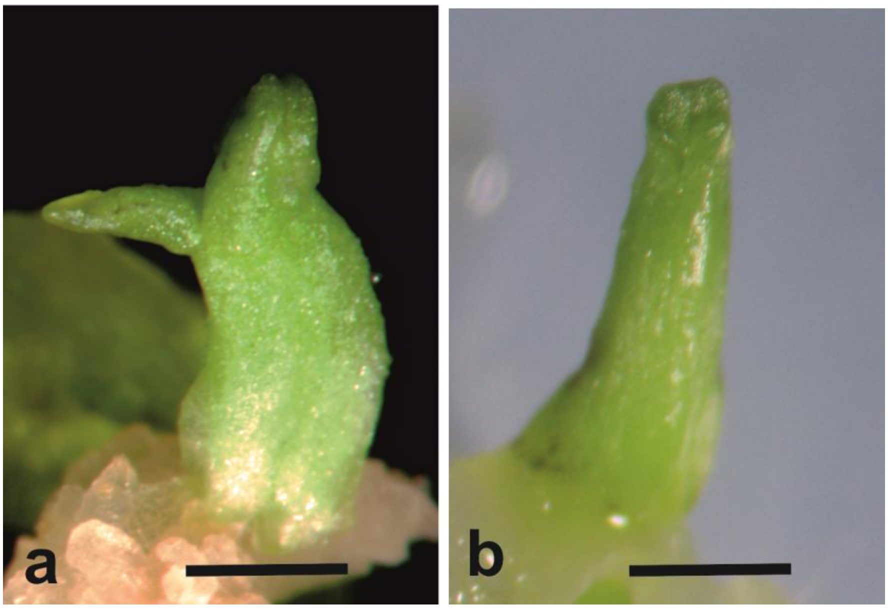

Supplement: Supplementary file 3 — (PDF 218 kb) [file 709_2015_814_MOESM3_ESM.pdf]
